# The ORF6 accessory protein contributes to SARS-CoV-2 virulence and pathogenicity in the naturally susceptible feline model of infection

**DOI:** 10.1128/jvi.00644-25

**Published:** 2026-02-27

**Authors:** Mohammed Nooruzzaman, Salman L. Butt, Ruchi Rani, Chengjin Ye, Andrew D. Miller, Luis Martinez-Sobrido, Ying Fang, Diego G. Diel

**Affiliations:** 1 Department of Population Medicine and Diagnostic Sciences, College of Veterinary Medicine, Cornell University 5922, Ithaca, New York, USA; 2 Texas Biomedical Research Institute 7075, San Antonio, Texas, USA; 3 Department of Pathobiology, College of Veterinary Medicine, University of Illinois at Urbana-Champaign 14589, Urbana, Illinois, USA; Loyola University Chicago - Health Sciences Campus, Maywood, Illinois, USA

**Keywords:** ORF6, virulence, pathogenesis, SARS-CoV-2

## Abstract

In this study, the infection dynamics, replication, and pathogenicity of a recombinant virus containing a deletion of ORF6 (rWA1ΔORF6) on the backbone of the highly virulent SARS-CoV-2 WA1 virus (rWA1) were investigated and compared to the parental rWA1 virus. While both rWA1 and rWA1ΔORF6 viruses replicated efficiently in cultured cells, the rWA1ΔORF6 virus produced smaller plaques, suggesting reduced cell-to-cell spread. Luciferase reporter assays revealed immune-suppressing effects of ORF6 on interferon (IFN) and nuclear factor kappa B (NF-κB) signaling pathways. Pathogenesis assessment in cats revealed that animals inoculated with rWA1 were lethargic and presented with fever on days 2 and 4 post-infection (pi), whereas rWA1ΔORF6-inoculated animals developed subclinical infection. Additionally, animals inoculated with rWA1ΔORF6 presented reduced infectious virus shedding in nasal and oral secretions and broncho-alveolar lavage fluid when compared with the rWA1-inoculated cats. Similarly, the rWA1ΔORF6-inoculated cats presented reduced virus replication in the respiratory tract as evidenced by lower viral loads and reduced lung inflammation on days 3 and 5 pi when compared to rWA1-inoculated animals. Host gene transcriptomic analysis revealed distinct differentially expressed gene (DEG) profiles in the nasal turbinate of animals infected with rWA1 when compared to rWA1ΔORF6. Importantly, type I IFN signaling was significantly upregulated in rWA1ΔORF6-infected cats when compared to rWA1-inoculated animals, which could potentially contribute to the reduced replication of rWA1ΔORF6 in the upper and lower respiratory tracts of infected animals. Collectively, these results demonstrate that the SARS-CoV-2 ORF6 is an important virulence determinant of the virus, contributing to the modulation of host antiviral immune responses.

**IMPORTANCE:**

SARS-CoV-2 encodes several proteins that inhibit host IFN responses. The accessory protein ORF6 antagonizes IFN signaling by blocking the nucleocytoplasmic trafficking of key transcription factors. In this study, we showed that ORF6 plays an important role in SARS-CoV-2 pathogenesis. While both rWA1 and rWA1ΔORF6 viruses replicated efficiently in cell culture, the rWA1ΔORF6 presented impaired cell-to-cell spread and reduced innate immune inhibition compared to the parental rWA1. A pathogenesis study in the feline model revealed an attenuated phenotype of the rWA1ΔORF6, indicating that ORF6 is a major virulence determinant of SARS-CoV-2.

## INTRODUCTION

The coronavirus disease 2019 (COVID-19) pandemic, caused by the severe acute respiratory syndrome coronavirus 2 (SARS-CoV-2), emerged in Wuhan, China, in late 2019. It has caused more than 779 million human infections, with more than 7.1 million deaths reported to the World Health Organization as of January 11, 2026 (1). Despite significant breakthroughs in vaccine, immunotherapeutic, and antiviral research and development, SARS-CoV-2 remains a major global health burden as variants of the virus continue to emerge. The clinical presentation of COVID-19 varies from asymptomatic infections to severe clinical disease and death, which affects both healthy individuals as well as individuals with underlying comorbidities (e.g., immunosuppression, cardiac diseases, and cancers) and involves imbalanced host innate immune responses (cytokine storm) (2).

Optimal induction of host type I interferon (IFN-I) responses is crucial to COVID-19 clinical outcomes, as dysregulated activation of IFN-I signaling pathways can be life-threatening (3, 4). IFN-I responses initiate through recognition of SARS-CoV-2 and its replication products (e.g., ssRNA and dsRNA) through pattern recognition receptors (PRRs) (5). Multiple cytosolic PRRs recognize SARS-CoV-2 RNA (RIG-I, MDA5, and TLR3) and host mitochondrial DNA (STING) and activate innate antiviral signaling cascades (6–10). Most of these signaling pathways converge and catalyze the phosphorylation and nuclear translocation of key transcription factors such as interferon regulatory factor 3 (IRF3), IRF7, and nuclear factor kappa B (NF-κB), and the subsequent transcription of IFN-I and IFN-III and proinflammatory cytokines. The secreted IFNs bind to their receptors to activate Janus kinase/signal transducers and activator of transcription (JAK-STAT) signaling pathway to drive the expression of IFN-stimulated genes (ISGs). These ISGs initiate an antiviral state in infected and bystander cells by suppressing critical steps of the viral replication cycle to impair virus spread. Additionally, the expression of ISGs can lead to the activation of immune cells and induce apoptosis of infected cells (11). To counteract host innate antiviral immunity, SARS-CoV-2 has evolved a plethora of mechanisms to inhibit IFN induction and subsequent signaling events. Several viral proteins, such as non-structural protein NSP1, NSP13, NSP14, and accessory proteins ORF3a, ORF6, and ORF9b have been shown to contribute to innate immune evasion (12–22).

SARS-CoV-2 accessory protein ORF6 is uniquely encoded by members of the *Sarbecovirus* subgenus of Betacoronaviruses. It is a polypeptide with 61 amino acids in length, which functions primarily to antagonize the host IFN signaling pathways, thus limiting host innate immune responses to IFN (13–17). The ORF6 protein interacts directly with nucleopore complex components nucleoporin 98 (NUP98) and ribonucleic acid export 1 (RAE1), which suppresses the nuclear import of key transcriptional molecules such as IRF3, STAT1, and STAT2 and inhibits the export of ISGs mRNAs (13, 16, 17, 20, 23, 24). A recent study showed that the ORF6 protein also interacts with RIG-I and inhibits early IFN induction (14).

A hallmark of SARS-CoV-2 is its ability to infect and replicate in a diverse group of animal species. While the function of ORF6 on host immune evasion has been investigated *in vitro*, studies on the role of ORF6 in SARS-CoV-2 pathogenesis are limited to K18 human angiotensin-converting enzyme 2 (hACE2) transgenic mouse and hamster models (13, 21). In this study, we investigated the role of the ORF6 protein using recombinant viruses with ORF6 deletion. The role of the ORF6 protein in virus replication and cell-to-cell spread and innate immune evasion was initially characterized *in vitro*, and the infection dynamics, tissue tropism, pathology, and host transcriptional responses were subsequently assessed using the highly susceptible feline model of SARS-CoV-2 infection.

## RESULTS

### The ORF6-deletion virus shows efficient replication but a reduced plaque size phenotype *in vitro*

**Fig 1:**
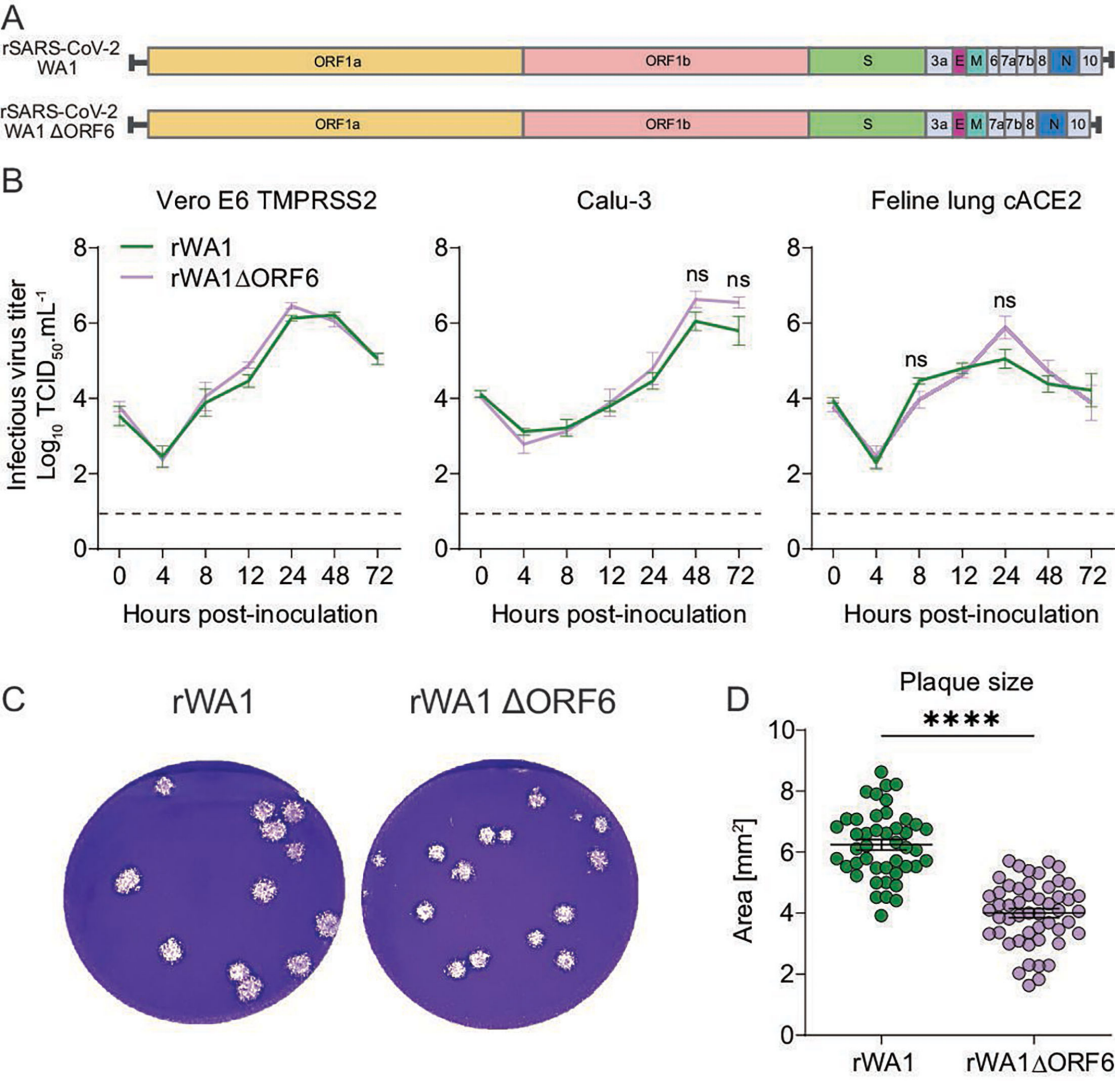
Role of SARS-CoV-2 accessory protein ORF6 in virus replication and spread *in vitro*. (**A**) Schematic representation of parental rSARS-CoV-2 WA1 (rWA1) and ORF6-deleted (rWA1ΔORF6) viruses used in this study. (**B**) Multi-step growth curves. Vero E6 TMPRSS2, Calu-3, and feline lung cACE2 cells were infected (MOI 0.1) with rWA1 and rWA1ΔORF6 viruses and incubated at 4°C for 1 h (virus adsorption) and then transferred and incubated at 37°C. Cells were harvested at the indicated time points, and the virus titers were determined by limiting dilutions and expressed as TCID_50_.mL^−1^. The limit of detection (LOD) for infectious virus titration was 10^1.05^ TCID_50_.mL^−1^, which is marked with the dashed line. Data are the mean ± SEM of three replicates (*n* = 3) from three independent experiments and two-way ANOVA with Tukey’s multiple comparison test, ns = not significant. (**C**) Plaque phenotype. Vero E6 cells were infected with rWA1 and rWA1ΔORF6 and overlaid with media containing 0.5% agarose. Plates were incubated at 37°C for 72 h, the agarose overlay was removed, cells were fixed, and the monolayer was stained with 0.5% crystal violet. Representative images of plaque phenotypes from three independent experiments are shown. (**D**) Viral plaque sizes. The diameters of viral plaques were measured using a scale in millimeters. Data indicate means ± SEM, *n* = 45, and two independent experiments. Mann-Whitney U test, **** *P* ≤ 0.0001.

To study the contribution of SARS-CoV-2 accessory protein ORF6 in virus infection, replication, and pathogenesis, we generated a recombinant virus deficient in ORF6 using reverse genetics (25). Using the rWA1 strain as a backbone, a recombinant SARS-CoV-2 lacking the ORF6 gene (rWA1ΔORF6) was generated and rescued (Fig. 1A). Complete genome sequencing of the recombinant virus confirmed ORF6 gene deletion and lack of unintended mutations in viral genomes. We next performed multi-step growth kinetics analysis to assess the replication efficiency of both rWA1 and rWA1ΔORF6 viruses using Vero E6 TMPRSS2, Calu-3, and feline lung cells stably expressing the cat ACE2 receptor (Feline lung cACE2). Both rWA1 and rWA1ΔORF6 viruses showed comparable replication kinetics in all three cell lines (Fig. 1B), indicating that deletion of ORF6 does not affect virus replication *in vitro*. Next, we assessed the effect of ORF6 deletion on cell-to-cell spread of the virus using plaque assays in Vero E6 cells. The rWA1ΔORF6 virus produced smaller (*P* ≤ 0.0001) plaque size compared to the parental rWA1 (Fig. 1C and D), suggesting the reduced ability of rWA1ΔORF6 for cell-to-cell spread. These results demonstrate that ORF6 deletion from the SARS-CoV-2 genome does not affect virus replication but leads to reduced cell-to-cell spread *in vitro*.

### SARS-CoV-2 accessory protein ORF6 inhibits multiple innate immune pathways *in vitro*

**Fig 2:**
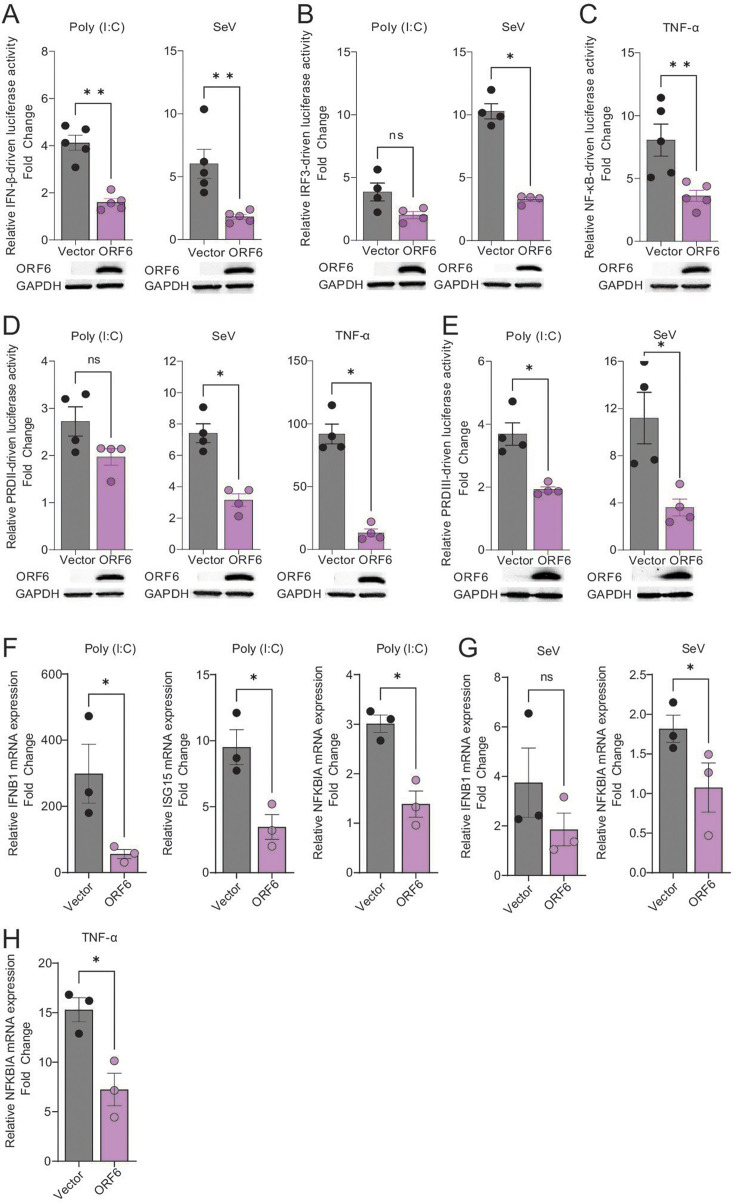
Innate immune inhibition by SARS-CoV-2 accessory protein ORF6. HEK293T cells were transfected with plasmids encoding ORF6 or an empty vector and with IFN-β (**A**), IRF3 (**B**), NF-κB (**C**), PRDII (**D**), and PRDIII (**E**) Firefly reporter plasmids. At 24 h post-transfection, the cells were stimulated with poly(I:C), SeV, or TNF-α for 12 h. Cell lysates were harvested, and Firefly luciferase activity was measured using a luminometer. The ratio of luminescence obtained from target reporters (IFN-β-Luc, IRF3-Luc, NF-κB-Luc, PRDII-Luc, and PRDIII-Luc) to luminescence from the control Renilla reporter (pRN-Luc) was determined to normalize for transfection efficiencies. The relative IFN-β-, IRF3-, NF-κB-, PRDII-, or PRDIII-driven luciferase activity was calculated as fold change over the unstimulated cells. HEK293T cells were transfected with plasmids encoding ORF6 or an empty vector and stimulated with poly(I:C) (**F**), SeV (**G**), or TNF-α (**H**) for 4 h. Cell lysates were harvested and analyzed by qPCR. (**A–H**) Data indicate mean ± SEM of 3–5 independent experiments. Mann-Whitney U test, * *P* ≤ 0.05, ns = not significant. One-way ANOVA with Tukey’s multiple comparison tests, ** *P* ≤ 0.01, *** *P* ≤ 0.001.

We investigated the role of SARS-CoV-2 ORF6 protein in modulating the host innate antiviral pathways *in vitro* by using luciferase reporter assays and confirmed this by gene expression analysis. First, we evaluated the inhibitory effect of ORF6 on the activation of IFN-β-, IRF3-, and NF-κB-driven promoters. Additionally, we investigated the inhibitory effect of ORF6 on two positive regulatory domains (PRDs) of the IFN-β enhanceosome, PRDII and PRDIII, that are recognized by transcription factors NF-κB and IRF3/IRF7, respectively (26). For this, we co-transfected HEK293T cells with plasmids encoding the ORF6 protein or an empty vector, with IFN-β-, IRF3-, NF-κB-, PRDII-, and PRDIII-driven Firefly luciferase reporter plasmids. A plasmid constitutively expressing Renilla luciferase reporter gene (pRN-Luc) was used as a control to normalize transfection efficiencies. At 24 h post-transfection, the cells were stimulated with Sendai virus (SeV) or poly(I:C) (two IFN inducers) or TNF-α (a NF-κB pathway inducer). After 12 h of stimulation, Firefly luciferase activity was quantified to assess the inhibitory role of ORF6 protein on these innate signaling pathways. Overexpression of ORF6 protein significantly downregulated the activation of poly(I:C)- and SeV-induced IFN-β signaling (Fig. 2A), SeV-induced IRF3 signaling (Fig. 2B), TNF-α-induced NF-κB signaling (Fig. 2C), SeV- and TNF-α-induced PRDII signaling (Fig. 2D), and poly(I:C)- and SeV-induced PRDIII signaling pathways (Fig. 2E).

To confirm the luciferase reporter assays, we performed gene expression analysis to evaluate the effect of ORF6 protein on innate immune inhibition (Fig. 2F through H). We transfected HEK293T cells with plasmids encoding ORF6 protein or an empty vector. At 24 h post-transfection, the cells were stimulated with poly(I:C), Sendai virus (SeV), or TNF-α as above. After 4 h of stimulation, the cell lysates were harvested and analyzed by qPCR. Upon poly(I:C) transfection, significant (*P* ≤ 0.05) downregulation of *IFNB1*, *ISG15,* and *NFKBIA* mRNA expressions was observed in cells overexpressing ORF6 protein compared to the empty vector control (Fig. 2F). Similarly, the expression of *NFKBIA* mRNA was also significantly downregulated in ORF6-overexpressing cells stimulated with SeV and TNF-α compared to the empty vector control (Fig. 2G and H). Together, these findings indicate that ORF6 antagonizes both IFN-β and NF-κB signaling pathways.

**Fig 3:**
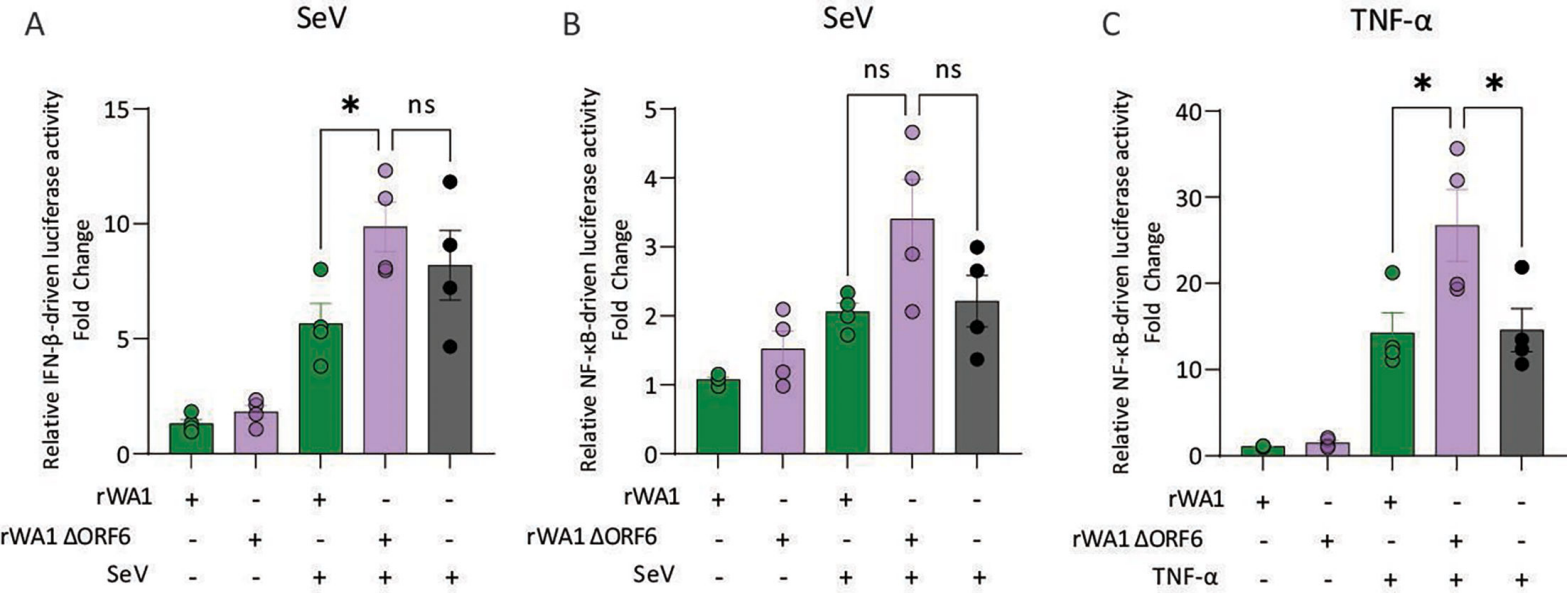
Role of ORF6 on innate immune inhibition during SARS-CoV-2 infection *in vitro*. HEK293T-hACE2 cells were transfected with IFN-β (**A**) or NF-κB (**B, C**) Firefly reporter plasmids for 6 h. Cells were then infected with rWA1 or rWA1ΔORF6 (MOI = 3) for 12 h. After that, the cells were stimulated with SeV or TNF-α for 8 h, cell lysates were harvested, and the luciferase activity was measured using a luminometer. Untreated control cells were harvested after 20 h of infection. The ratio of luminescence obtained from the target reporters (IFN-β-Luc or NF-κB-Luc) to luminescence obtained from the control Renilla reporter (pRN-Luc) was determined to normalize for transfection efficiencies. The relative IFN-β or NF-κB promoter-driven luciferase activity was expressed as fold change over the unstimulated cells. Data indicate mean ± SEM of four independent experiments. One-way ANOVA with multiple comparison test, * *P* ≤ 0.05, ns = not significant.

Next, we tested the relevance of the innate immune inhibitory effects of ORF6 overexpression in the context of SARS-CoV-2 infection. For this, HEK293T cells expressing the human ACE2 receptor (HEK293T-hACE2) were transfected with IFN-β- or NF-κB-Firefly luciferase plasmids for 6 h. The cells were then infected with rWA1 or rWA1ΔORF6 (MOI 3) for 12 h, followed by stimulation with SeV or TNF-α for 8 h. Cell lysates were harvested, and the luciferase activity was quantified as above. Infection of HEK293T-hACE2 cells with parental rWA1 or rWA1ΔORF6 viruses induced low-to-moderate IFN-β or NF-κB reporter activation (Fig. 3A through C). However, subsequent stimulation of the SARS-CoV-2-infected cells with SeV and TNF-α greatly enhanced the IFN-β and/or NF-κB promoter signaling, which was comparable to or higher than the SeV or TNF-α stimulation alone (Fig. 3A through C). Of note, SeV stimulation of rWA1ΔORF6-infected cells induced higher IFN-β promoter activation (*P*<0.05) than the rWA1-infected cells (Fig. 3A), suggesting that deletion of ORF6 from rWA1 virus resulted in impaired ability of the virus to inhibit the interferon signaling pathway. Similarly, TNF-α stimulation of the rWA1ΔORF6-infected cells also resulted in higher NF-κB promoter activation (*P*<0.05) compared to the TNF-α stimulated rWA1-infected cells (Fig. 3C). Collectively, these results highlight the important role of ORF6 in innate immune inhibition during SARS-CoV-2 infection.

### Infection with rWA1ΔORF6 leads to subclinical infection and reduced virus shedding in cats

**Fig 4:**
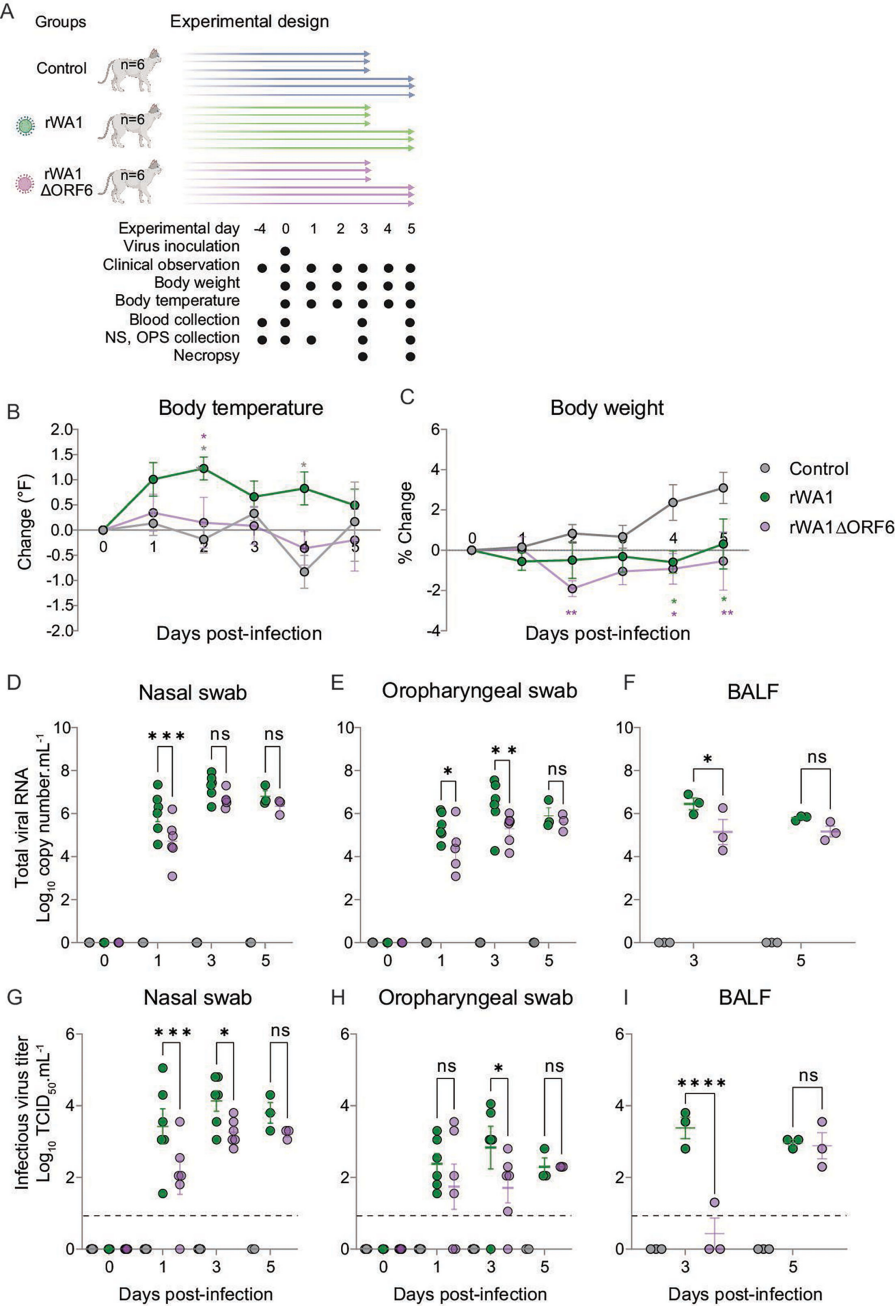
ORF6 contributes to SARS-CoV-2 virulence and pathogenesis in cats. (**A**) Experimental study design. (**B**) Changes (°F) in body temperature and (**C**) body weight (%) in cats intranasally inoculated with rWA1 and rWA1ΔORF6. SARS-CoV-2 RNA loads quantified by rRT-PCR in nasal (**D**) and oral (**E**) secretions collected on days 0, 1, 3, and 5 pi and in bronchoalveolar lavage fluid (**F**) collected on days 3 and 5 pi. Infectious SARS-CoV-2 loads in nasal (**G**) and oral (**H**) secretions and BALF (**I**) were determined by virus titration in rRT-PCR-positive samples. Virus titers were determined using endpoint dilutions and expressed as TCID_50_.mL^−1^. The limit of detection (LOD) for infectious virus titration was 10^1.05^ TCID_50_.mL^−1^, which is depicted with the dashed line. Data indicate the mean ± standard deviation of 3–6 animals per group per time point. Two-way ANOVA with Tukey’s multiple comparison test, ns = not significant, * *P ≤* 0.05; ** *P ≤* 0.01; *** *P ≤* 0.001; **** *P ≤* 0.0001.

After acclimation, nasal swabs and blood samples were collected from all cats and tested by rRT-PCR for SARS-CoV-2 and virus neutralization using SARS-CoV-2 B.1 and Omicron BA1.1 lineages, which confirmed that the animals were negative for SARS-CoV-2. Three groups of experimental cats were inoculated with rWA1 or rWA1ΔORF6 viruses or mock-infected (Fig. 4A). Clinical signs, virus replication in tissues, virus shedding, and pathology in respiratory tissues were evaluated (Fig. 4A). Cats inoculated with rWA1 were depressed and presented elevated body temperature throughout the 5-day experimental period with significant increase in the body temperature being observed on day 2 post-infection (pi) (*P* ≤ 0.05), when compared to rWA1ΔORF6- and mock-inoculated groups (Fig. 4B). No clinical sign was observed in rWA1ΔORF6- and mock-inoculated cats. Both virus-inoculated groups lost or maintained body weight, while control cats gained body weight throughout the study period (Fig. 4C).

We next assessed the dynamics of virus shedding in inoculated cats. To this end, nasal (NS) and oropharyngeal (OPS) swabs were collected from control and inoculated cats on days 0, 1, 3, and 5 pi, and bronchoalveolar lavage fluid (BALF) was collected on days 3 and 5 pi at necropsy and tested by rRT-PCR and endpoint virus titrations. Overall, viral RNA loads in NS, OPS, and BALF were lower in rWA1ΔORF6-inoculated cats when compared to rWA1-inoculated animals throughout the experiment. The rWA1ΔORF6-inoculated cats presented significantly lower viral RNA in nasal swabs on day 1 pi (*P* ≤ 0.001) than rWA1-inoculated animals (Fig. 4D). Similarly, lower viral RNA levels were found in OPS of rWA1ΔORF6-inoculated cats when compared to rWA1-inoculated animals on days 1 (*P* ≤ 0.05) and 3 (*P* ≤ 0.01) pi (Fig. 4E). Consistent with virus replication in the upper respiratory tract, lower viral RNA loads were detected in BALF of rWA1ΔORF6-inoculated cats when compared to rWA1-inoculated animals on day 3 pi (*P* ≤ 0.05) (Fig. 4F).

To study the dynamics of infectious virus shedding in NS, OPS, and BALF, we quantified infectious viruses in rRT-PCR-positive swab samples by endpoint virus titrations. Similar to the rRT-PCR results, infectious virus titers in NS, OPS, and BALF were lower in rWA1ΔORF6-inoculated cats than in rWA1-inoculated animals (Fig. 4G through I). Infectious virus titers in NS were significantly lower in rWA1ΔORF6-inoculated cats on days 1 (0–2.55 log_10_ TCID_50_.mL^−1^ vs 1.55–5.05 log_10_ TCID_50_.mL^−1^, *P* ≤ 0.001) and 3 pi (2.8–3.8 log_10_ TCID_50_.mL^−1^ vs 3.05–4.8 log_10_ TCID_50_.mL^−1^, *P* ≤ 0.05) when compared to rWA1-inoculated animals (Fig. 4G). The infectious virus titers in OPS were about 1 log_10_ lower than the virus titers obtained in NS. Similarly, rWA1ΔORF6-inoculated cats had a lower viral load in OPS on day 3 pi (*P* ≤ 0.05) than rWA1-inoculated animals (Fig. 4H). In BALF, infectious virus was isolated from all three cats inoculated with rWA1 virus on day 3 pi, while only one cat from the rWA1ΔORF6-inoculated group presented infectious virus in BALF (Fig. 4I). The BALF from all inoculated cats on day 5 pi showed positive virus isolation and a comparable virus titer between rWA1- and rWA1ΔORF6-inoculated animals (Fig. 4I). Together, these results demonstrate that deletion of ORF6 from the SARS-CoV-2 WA1 genome resulted in subclinical infection and reduced virus replication and shedding in cats. Thus, ORF6 contributed to SARS-CoV-2 virulence and pathogenesis in the naturally susceptible feline model of SARS-CoV-2 infection.

### Deletion of ORF6 from SARS-CoV-2 WA1 results in limited virus replication in respiratory tissues

**Fig 5:**
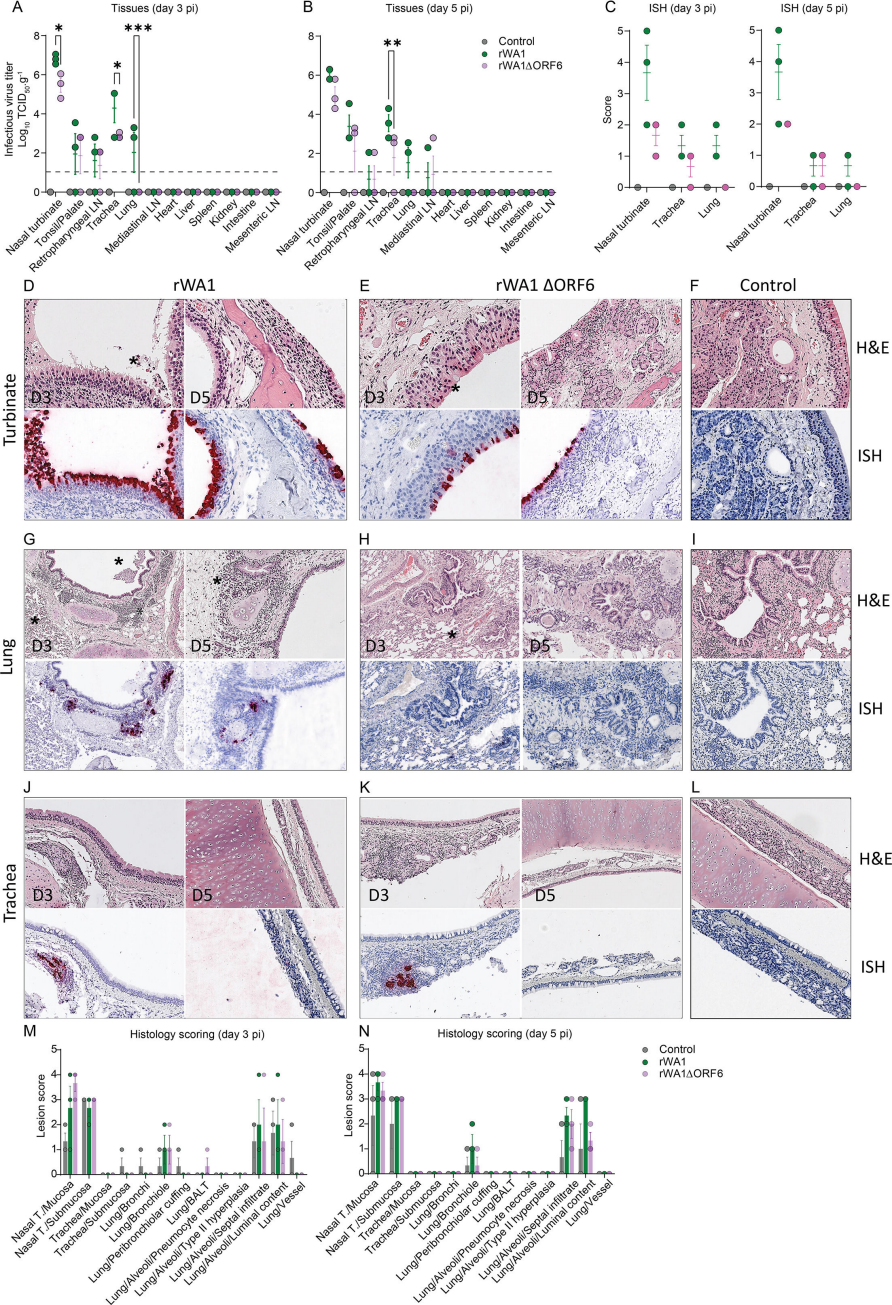
The rWA1ΔORF6 virus presents reduced replication and pathology in the respiratory tract of cats. Infectious SARS-CoV-2 in tissues was assessed by virus titration in rRT-PCR-positive tissue samples collected on days 3 (**A**) and 5 (**B**) pi. Virus titers were determined using endpoint dilutions and expressed as TCID_50_.mL^−1^. The limit of detection (LOD) for infectious virus titration was 10^1.05^ TCID_50_.mL^−1^, which is depicted with the dashed line. Data indicate mean ± standard deviation of 3 animals per group per time point. (**C**) *In situ* hybridization (ISH) scoring of tissues. (**A–C**) Two-way ANOVA with Tukey’s multiple comparison test, * *P ≤* 0.05; ** *P ≤* 0.01; *** *P ≤* 0.005. Hematoxylin and eosin (H&E) staining and ISH in the nasal turbinate (**D–F**), trachea (**G–I**), and lung (**J–L**) of cats inoculated with rWA1 and rWA1ΔORF6 and control cats. Nasal turbinates, trachea, and lungs were collected from rWA1 (**D, G, and J**) and rWA1ΔORF6 (**E, H, and K**) or mock (**F, I, and L**) inoculated cats on days 3 and 5 pi. In ISH, the viral RNA (red labeling) was performed using a probe targeting the SARS-CoV-2 S RNA. Lesion scores (established as described in Materials and Methods) for the tissues evaluated on days 3 and 5 pi are presented in (**M**) and (**N**), respectively.

We studied the effect of ORF6 deletion on tissue tropism and replication of SARS-CoV-2. For this, tissues collected from rWA1- and rWA1ΔORF6-inoculated cats were tested for the presence of the virus by titrations and *in situ* hybridization (ISH). Tissue samples, including nasal turbinate, tonsil/palate, retropharyngeal lymph node, trachea, lungs, mediastinal lymph node, heart, liver, spleen, kidney, intestine, and mesenteric lymph node, were collected at necropsy on days 3 and 5 pi from control and inoculated cats and processed for infectious virus quantification. The highest infectious virus titers were detected in nasal turbinates from rWA1-inoculated cats on day 3 pi (titers range: 6.55–7.05 log_10_ TCID_50_.g^−1^), while significantly lower virus titers were obtained from rWA1ΔORF6-inoculated cats (titers range: 2.8–6.05 log_10_ TCID_50_.g^−1^) (*P* ≤ 0.05) (Fig. 5A). Similarly, on day 5 pi, rWA1ΔORF6-inoculated cats had lower virus titers in nasal turbinates (titer range: 4.3–5.8 log_10_ TCID_50_.g^−1^) than rWA1-inoculated cats (titers range: 5.8–6.3 log_10_ TCID_50_.g^−1^) (Fig. 4B). The rWA1ΔORF6-inoculated cats (titers range: 0–2.8 log_10_ TCID_50_.g^−1^) also had significantly lower virus titers in trachea (*P* ≤ 0.01) on day 5 pi when compared to rWA1-inoculated cats (titers range: 2.8–4.3 log_10_ TCID_50_.g^−1^) (Fig. 5A and B). No infectious virus was detected in tissues collected from the control cats.

We next localized virus replication sites in tissues of inoculated cats using ISH and scored the relative viral RNA labeling in tissues following a scoring system described previously (27). For this, we stained the nasal turbinate, trachea, and lungs collected from all animals on days 3 and 5 pi for SARS-CoV-2 RNA using RNAscope ZZ technology. Viral RNA labeling score showed significantly (*P* ≤ 0.05) higher labeling in the nasal turbinates and lungs of rWA1-inoculated cats on day 3 pi and in nasal turbinates on day 5 pi compared to the rWA1ΔORF6-inoculated animals (Fig. 5C). The epithelial cells of nasal mucosa were the predominant cell types showing positive labeling for SARS-CoV-2 RNA with intense hybridization being observed on day 3 pi, which was slightly less abundant on day 5 pi. The nasal turbinates collected from rWA1-inoculated cats showed more intense labeling for SARS-CoV-2 RNA on days 3 and 5 pi when compared to rWA1ΔORF6-inoculated animals (Fig. 5C and D). All SARS-CoV-2-inoculated cats from both groups showed positive viral RNA hybridization in nasal turbinates; however, the intensity and number of positive cells were higher in rWA1-inoculated cats when compared with rWA1ΔORF6-inoculated animals. In the trachea, localized viral staining was detected in cells within submucosal interstitial stroma, as well as cells associated with submucosal glandular or vascular elements (Fig. 5G and H). Viral RNA hybridization in the trachea was also higher in rWA1-inoculated cats on both days 3 and 5 pi compared to rWA1ΔORF6-inoculated animals. In the lungs, intense viral RNA hybridization was mostly observed in bronchial glandular epithelial cells with sparse staining of bronchial epithelial cells of rWA1-inoculated cats on both days 3 (2 cats) and 5 (1 cat) pi (Fig. 5F). No viral RNA labeling was found in the lungs of rWA1ΔORF6-inoculated cats on either day 3 or 5 pi (Fig. 5C and G). Tissues collected from control cats remained negative for viral RNA using ISH (Fig. 5F, I, and L).

### Deletion of ORF6 from the SARS-CoV-2 WA1 virus genome results in limited lung pathology in cats

The histological changes in the nasal turbinate, trachea, and lungs of cats were evaluated and scored on days 3 and 5 pi (Fig. 5). In the nasal turbinate, loss of mucosal epithelial cells and mild-to-moderate epithelial necrosis were noticed in both rWA1 and rWA1ΔORF6 inoculated cats on days 3 and 5 pi (Fig. 5D, E, M, and N). Aggregates of fibrin, necrotic debris, and inflammatory cells in the nasal passages were also observed in both inoculated groups. No remarkable histological changes were observed in the trachea (Fig. 5G, H, M, and N). In the lungs, bronchiolar necrosis and mixed inflammation with exudates in the lumen were observed in two rWA1- and rWA1ΔORF6-inoculated cats on day 3 pi and two rWA1- and one rWA1ΔORF6-inoculated cat on day 5 pi (Fig. 5J, K, M, and N). Thickening of the alveolar septa due to mononuclear infiltration was noticed in all three rWA1-inoculated cats, but in one rWA1ΔORF6-inoculated animal on day 3 pi. Alveolar septal thickening was also noticed in all rWA1- and rWA1ΔORF6-inoculated cats on day 5 pi (Fig. 5J, K, M, and N). Exudates in the alveolar lumen were found in most of the inoculated cats on both days 3 and 5 pi, with lower severity in rWA1ΔORF6-inoculated cats. The control cats had normal histological architectures in all tissues studied (Fig. 5F, I, M, and N). Collectively, these results demonstrate that the rWA1ΔORF6 virus induces limited pathology in the lungs of cats.

### Deletion of ORF6 from the SARS-CoV-2 WA1 virus genome results in a differential transcriptional profile in the nasal turbinates of cats

**Fig 6:**
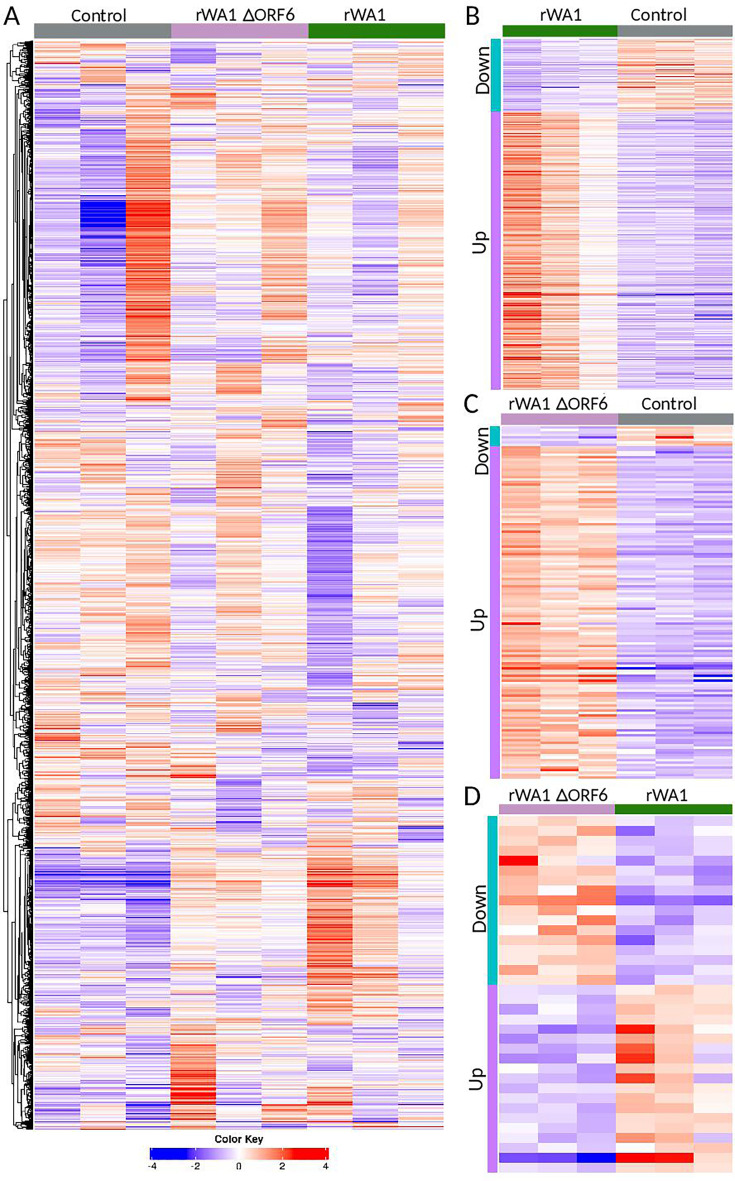
Heatmaps of differential gene expression in the nasal turbinate of cats infected with SARS-CoV-2. Nasal turbinate was collected from rWA1 and rWA1ΔORF6 or mock-inoculated cats on day 3 pi and subjected to RNA-seq analysis. (**A**) Heatmap of hierarchically clustered DEGs in nasal turbinate tissues of mock- (control), rWA1ΔORF6- and rWA1-infected cats. Each column represents the gene expression profiles from individual samples across three groups. A total of 5,000 genes represented by colored bars represent z-score values in the color key, where red represents upregulation, blue represents downregulation, and white represents unchanged gene expression. A group comparison of DEGs in rWA1 vs. control showed 394 genes up- and 104 down-regulated genes (**B**), 134 up- and 8 down-regulated genes in rWA1ΔORF6 vs. control (**C**), and 19 up- and 17 down-regulated genes in rWA1ΔORF6 vs. rWA1 groups (**D**). The up- and down-regulated DEGs are clustered across three samples from each group.

We performed bulk RNA-seq to investigate the differential expression of genes in nasal turbinates (the first site of SARS-CoV-2 replication and where we observed the highest viral loads) collected from control or from rWA1- and rWA1ΔORF6-infected cats. Differential gene expression analysis revealed several thousand differentially expressed host genes (DEGs). The relative gene expression obtained by comparing rWA1ΔORF6 vs. control and rWA1 vs. control is summarized in the heatmap showing the top 5,000 hierarchically clustered DEGs across the three groups (Fig. 6A). Each column represents the gene expression profile from individual samples, and the colored bars (blue, white, and red bars represent z score values in the color key) represent upregulated (red), downregulated (blue), or unchanged (white) gene expression. A comparison of significant DEGs revealed a total of 394 up- and 104 down-regulated genes in rWA1 vs. control group (Fig. 6B), 134 up- and 8 down-regulated DEGs in rWA1ΔORF6 vs. control group (Fig. 6C), and 19 up- and 17 down-regulated DEGs in rWA1 vs. rWA1ΔORF6 groups (Fig. 6D).

### Gene set enrichment analysis (GSEA)

#### Cellular pathway enrichment

**Fig 7:**
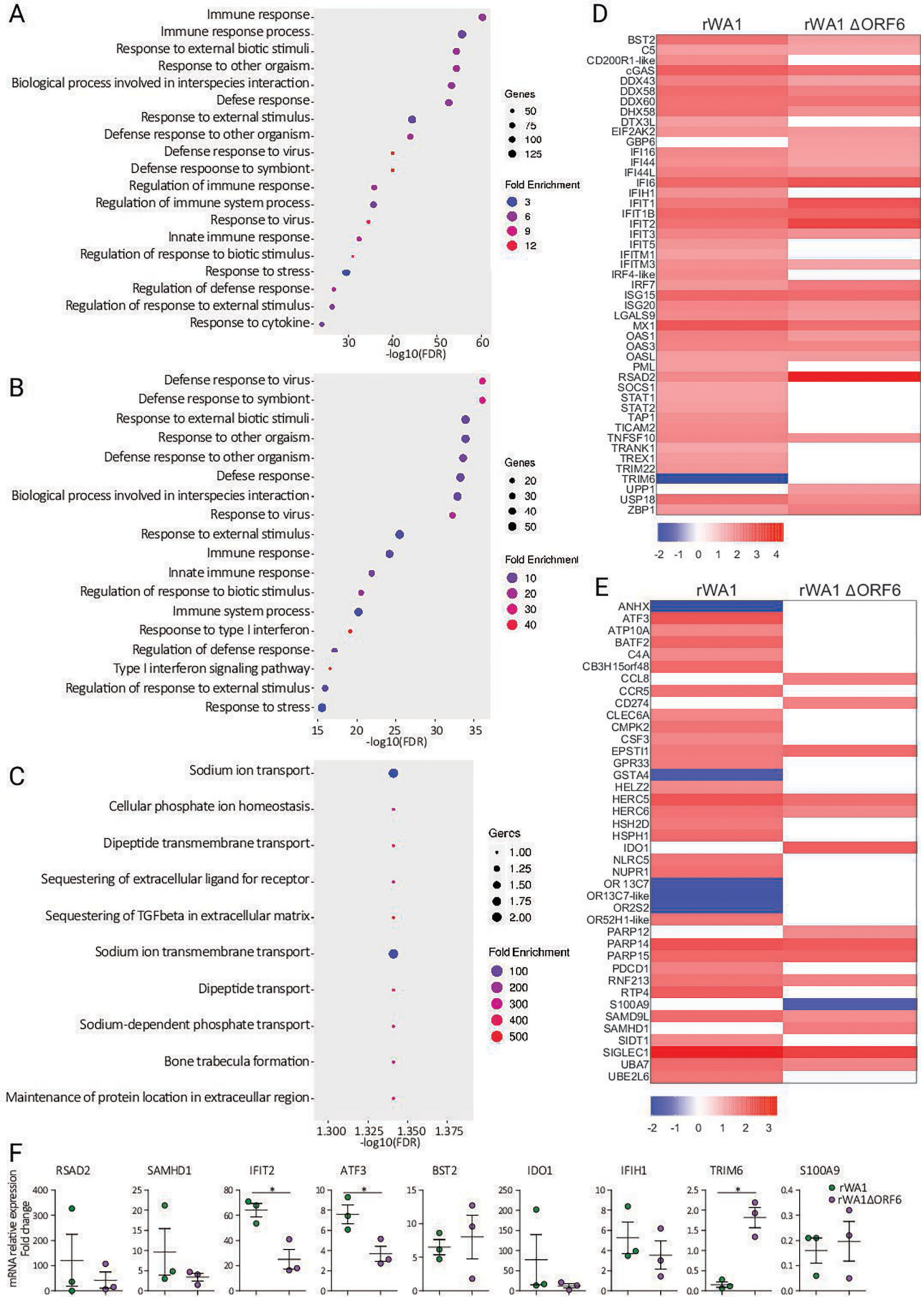
rWA1 and rWA1ΔORF6 trigger different cellular pathways in the nasal turbinate of cats. Nasal turbinate tissues were collected from rWA1 and rWA1ΔORF6 or mock-inoculated cats on days 3 pi and subjected to RNA-seq analysis. Dot plot showing the upregulated GO biological processes across three group comparisons, rWA1 vs. control (**A**), rWA1ΔORF6 vs. control (**B**), and rWA1ΔORF6 vs. rWA1 (**C**). Heatmaps showing log_2_FC expression profile of top 40 interferon-stimulated genes (ISGs) (**D**) and top 40 transcription factors (**E**) in the nasal turbinate of cats infected with rWA1 and rWA1ΔORF6. (**F**) Dot plots showing verification of gene expression (qPCR) of the top nine differentially expressed genes in the nasal turbinate of cats. Data indicate mean ± SEM of 3 animals per group. Mann-Whitney U test, *P* ≤ 0.05 indicates statistical significance.

The significant DEGs from the three groups (control, rWA1, and rWA1∆ORF6) were further used for pathway enrichment analysis, and the top significantly enriched GO biological pathways are presented as dot plots in Fig. 7A through C. This analysis revealed that the rWA1 and rWA1ΔORF6 triggered different pathways in the nasal turbinate of infected cats. The significantly enriched pathways were related to innate immune response in both rWA1- and rWA1ΔORF6-infected cats. Overall, the fold enrichment of innate immune response pathways was much higher in rWA1ΔORF6 relative to rWA1-infected cats. In rWA1-infected cats, the antiviral signaling pathways were 12-fold enriched, and the stress-response pathway was enriched 3-fold (Fig. 7A). In contrast, in rWA1ΔORF6-infected cats, the type I IFN signaling pathway was enriched 40-fold, and the response to type I IFN pathway was enriched 27-fold when compared to rWA1-inoculated cats (Fig. 7B).

Next, we investigated the association of the presence or absence of ORF6 in rWA1- and rWA1ΔORF6-infected cats on the host innate immune response, especially on ISGs expression, and their transcription factors. To investigate the differences in innate immune response to rWA1 and rWA1ΔORF6, we selected 80 significant DEGs associated with innate immune response from groups of rWA1 vs. control and rWA1ΔORF6 vs. control groups. Their relative fold changes are summarized in the heatmap presented in Fig. 7D and E. Notably, ISGs such as *BST2*, *Mx1*, *IFI6*, and *cGAS* were upregulated, and *TRIM6* was downregulated in rWA1 when compared to rWA1ΔORF6-infected cats. Expression of *GBP6* and *UPP1* was unchanged in rWA1-infected cats, while these genes were upregulated in rWA1ΔORF6-inoculated animals. Similarly, *RSAD2*, *IFIT1*, *IFIT2*, and *IRF7* were upregulated, while the expression of several ISGs (*IFIH1*, *IFIT5*, *IFITM1*, *IRF4-like*, *PML*, *SOCS1*, *STAT1*, *STAT2*, *TRIM22*, *TRIM6*, *TRAM1*, and *TREX1*) remained unchanged in rWA1ΔORF6-infected cats, compared to rWA1-infected cats where these genes were upregulated. Among the transcription factors, genes associated with olfactory transduction (*OR13C-7* and *OR2S2*), *ANHX*, and *GSTA4* were downregulated in rWA1-infected cats, while their expression remained unchanged in rWA1ΔORF6-infected cats. Additionally, *CCL8*, *CD274*, *IDO1*, *PARP12*, *S100A9*, and *SAMHD1* expression remained unchanged in rWA1-infected cats, whereas these genes were upregulated in rWA1ΔORF6-infected cats (Fig. 7E).

We selected the top nine differentially expressed genes from all three comparison panels, rWA1 vs. control, rWA1ΔORF6 vs. control, and rWA1ΔORF6 vs. rWA1 for the verification of gene expression by rRT-PCR (Fig. 7F). The *IFIT2* and *ATF3* genes were significantly (*P* ≤ 0.05) upregulated in rWA1 compared to the rWA1ΔORF6-inoculated animals, whereas *TRIM6* was significantly (*P* ≤ 0.05) downregulated in rWA1 compared to rWA1ΔORF6-inoculated animals. Other genes such as *RSAD2*, *SAMHD1*, *BST2*, *IDO1*, *IFIH1*, and *S100A9* were upregulated but remained comparable between both rWA1- and rWA1ΔORF6-inoculated animals (Fig. 7F).

## DISCUSSION

Our study builds on previous work (21) demonstrating that the SARS-CoV-2 accessory protein ORF6 is a bona fide virulence determinant that contributes to disease pathogenesis by modulating host innate immune responses. Using a highly relevant and naturally susceptible feline model of SARS-CoV-2 infection (27–34), we demonstrated that deletion of ORF6 from the SARS-CoV-2 WA1 genome results in an attenuated disease phenotype. The rWA1∆ORF6-infected animals present subclinical infection and markedly reduced virus replication and associated pathology in the upper and lower respiratory tracts.

The SARS-CoV-2 ORF6 protein functions to counteract host innate immune responses by selectively blocking the nuclear import and translocation of IRF3 and STAT transcription factors (13, 16, 17, 23, 24, 35). To further investigate the inhibition of downstream IFN signaling pathways by ORF6 protein, we used luciferase reporter assays. In line with previous studies, transient overexpression of ORF6 protein significantly downregulated Sendai virus (SeV)-induced IFN-β and IRF3 promoter activation (13, 14). However, we also found a significant inhibition of TNF-α-induced NF-κB promoter activation upon ORF6 overexpression, which differs from an earlier study (13). We further investigated the IFN-β promoter activity and included two positive regulatory domains (PRDs) of the IFN-β enhanceosome, PRDII and PRDIII, that are recognized by transcription factors NF-κB and IRF3/IRF7, respectively (26). The ORF6 protein also inhibited SeV- and TNF-α-induced PRDII and poly(I:C) and SeV-induced PRDIII promoter activation. To address the relevance of these findings in the context of infection, we infected HEK293T-hACE2 cells with rWA1 and rWA1ΔORF6 viruses and quantified the activation of both IFN-β and NF-κB reporters. Infection of HEK293T-hACE2 cells with recombinant viruses alone induced low IFN-β and NF-κB activation, which increased significantly upon subsequent stimulation with SeV and/or TNF-α. Of note, compared to the parental rWA1, stimulation of rWA1ΔORF6-infected cells with SeV and TNF-α slightly enhanced IFN-β and NF-κB-promoter activation, respectively. The modest stimulation of IFN-β- and NF-κB-mediated gene expression in the context of rWA1ΔORF6 infection could be a result of redundant functions of other innate immune inhibitors encoded by SARS-CoV-2, whose function during infection could compensate for the absence of ORF6.

Experimental animal studies evaluating the role of the ORF6 protein in SARS-CoV-2 pathogenicity are limited in previous reports. Using K18 human ACE2 transgenic mice, we demonstrated that 50% of the mice infected with ORF6-deficient virus survived while all wild-type virus-infected mice succumbed to infection (21). The reduced virulence of ORF6-deficient virus-infected mice was also mirrored in the lower virus loads in the nasal turbinate and lungs, as well as reduced lung lesion score, compared to wild-type virus-infected animals (21). Another study in Syrian golden hamsters inoculated with ORF6-deleted virus showed significantly reduced body weight loss and early recovery when compared to animals infected with wild-type virus, but similar virus titers were recovered from the nasal turbinates and lungs of both infected groups (13).

Here, we used a domestic cat model to study the role of ORF6 in SARS-CoV-2 pathogenesis. This animal model had previously reproduced several characteristics of the virus replication, clinical presentation, and respiratory lesions of COVID-19 disease in humans (27, 29, 36). In our current study, we demonstrated that cats inoculated with rWA1 were depressed and had an elevated body temperature; in contrast, rWA1ΔORF6-infected animals were subclinical and remained afebrile. Both inoculated groups, however, lost or maintained steady body weight, whereas the mock-infected cats gained body weight throughout the study. The rWA1ΔORF6-infected cats showed reduced virus replication in the nasal turbinate, trachea, and lungs and lower virus shedding in nasal and oropharyngeal swabs and bronchoalveolar lavage fluid, in comparison to rWA1-infected animals. Histological examination of lungs also revealed a reduced lung pathology in rWA1ΔORF6-infected cats, compared to rWA1-infected animals. Collectively, these findings showed an attenuated phenotype of rWA1ΔORF6 in cats, indicating a role for ORF6 in pathogenesis and the host range of SARS-CoV-2 in felids.

We further investigated the transcriptional response to rWA1 and rWA1ΔORF6 virus infection in the nasal turbinate of cats. The transcriptome of the nasal turbinates highlighted the processes underlying the attenuation of rWA1ΔORF6 SARS-CoV-2 virus during infection. We observed marked differences in DEGs in the nasal turbinates of cats infected with rWA1 and rWA1ΔORF6 viruses. This points to the fact that the ORF6 gene plays a significant role in viral pathogenesis. Studies have reported that the ORF6 gene disrupts IFN signaling by disrupting the nuclear translocation of IRF3 and STAT1 (15); however, Li and colleagues have reported otherwise that ORF6 is not sufficient to counteract IFN induction during viral infection (37). In this study, we found that type I IFN signaling was significantly upregulated in rWA1ΔORF6-infected cats compared to rWA1-infected cats, which is consistent with our *in vitro* cell culture experiments, as evidenced by the upregulation of IFN-β and IRF3 promoter activation in luciferase assays. This could be due to the significant upregulation of the master regulator of ISGs and RSAD2 in rWA1ΔORF6-infected cats compared to rWA1-infected cats.

Despite these findings, the magnitude of innate immune activation in nasal turbinate tissues from rWA1ΔORF6-infected cats was modest. One possible explanation is that day 3 post-infection may not represent the optimal time point to capture maximal differences in innate immune responses. Additionally, several ISGs were strongly induced in rWA1-infected cats, potentially reflecting the higher viral replication of the parental virus in the nasal turbinate, thereby eliciting more robust ISG responses than the attenuated rWA1ΔORF6 virus.

In summary, our study demonstrates the inhibitory role of ORF6 protein on host innate immune responses, and deletion of ORF6 attenuates SARS-CoV-2 pathogenesis. These findings will have important implications in coronavirus control and prevention.

## MATERIALS AND METHODS

### Cells and viruses

Vero E6 (ATCC CRL-1586) and Vero E6 TMPRSS2 (JCRB Cell Bank, JCRB1819) were propagated in Dulbecco’s-modified Eagle medium (DMEM), supplemented with 10% fetal bovine serum (FBS), L-glutamine (2 mM), penicillin (100 U.mL^−1^), streptomycin (100 μg.mL^−1^), and gentamicin (50 μg.mL^−1^). HEK293T cells were maintained in complete growth media consisting of minimum essential media (Corning, 10-010-CV), supplemented with 10% FBS, penicillin (100 U.mL^−1^), and streptomycin (100 μg.mL^−1^). HEK293T-hACE2 (BEI Resources, NR-52511) cells were grown in Dulbecco’s modified Eagle’s medium containing 4 mM L-glutamine, 4,500 mg per L glucose, 1 mM sodium pyruvate, and 1,500 mg/L sodium bicarbonate, supplemented with 10% fetal bovine serum. The cell cultures were maintained at 37°C with 5% CO_2_.

Sendai virus (SeV) (Cantell strain) was propagated in 11-day-old embryonated chicken eggs and titrated using a hemagglutination assay (HA).

The bacterial artificial chromosome (BAC) harboring the whole genome of the ancestral SARS-CoV-2 WA1 (rWA1) was generated as previously described (25). Deletion of accessory protein ORF6 and generation of recombinant SARS-CoV-2 deficient of ORF6 (rWA1ΔORF6) was described previously (21). For virus rescue, Vero E6 TMPRSS2 cells (3 × 10^5^ per well of a 6-well plate) were transfected with 2 µg/well of the respective SARS-CoV-2 BAC DNA (pBAC-WA1 or pBAC-WA1ΔORF6) using Lipofectamine 3000 (ThermoScientific, USA) and incubated at 37°C. Twenty-four hours later, the culture medium was replaced with complete growth medium supplemented with 5% FBS and incubated for an additional 48 h at 37°C. Following the development of cytopathic effects, infected cells and supernatants were harvested and labeled as P0. The recombinant viruses were propagated twice in Vero E6 TMPRSS2 cells. The whole genome sequences of the virus stocks were determined to confirm that no mutations occurred during rescue and amplification in cell culture. The titers of virus stocks were determined by plaque assays and end-point dilutions.

### Generation of feline lung cells stably expressing cat ACE2 receptor

A lentiviral plasmid encoding cat ACE2 (pscALPS-cACE2) was obtained from Addgene (#158082). A C-terminal Myc-tag epitope was added to facilitate detection. Lentiviral particles encoding the cACE2 receptor were produced in HEK293T cells. For this, 7.2 × 10^5^ cells were seeded in each well of a six-well plate. Twenty-four hours later, the cells were transfected with the lentiviral packaging vectors psPAX2 (1 µg, Addgene, 12260) and pMD2.G (1 µg, Addgene, 12259), together with the pscALPS-cACE2 transfer vector (1 µg). Lentiviral particles were harvested from the supernatant of transfected cells at 72 h post-transfection, cleared by centrifugation, aliquoted, and stored at −80°C until use.

Feline lung cells were seeded (7.2 × 10^5^ cells/well) in a six-well plate and incubated overnight. For transduction, the culture medium was removed; 1 mL of the lentivirus was added to each well and adsorbed for 2 h at 37°C with the plate rocking back and forth every 30 min. In addition, 2 mL of complete growth media was added to the cells and incubated for 72 h at 37°C. For selecting the transduced cells, the media were replaced with complete growth media containing 5 µg.mL^−1^ of puromycin dihydrochloride (Gibco, A1113803). The puromycin selection was continued until the complete death of non-transduced cells, and the expression of cACE2 was validated via immunofluorescence and immunoblots through the detection of the c-terminal Myc tag (Myc-Tag-9B11 Mouse mAb, Cell Signaling Technology, 2276).

### Viral replication kinetics

Viral growth kinetics of rWA1 and rWA1ΔORF6 were performed using three different cell lines: Vero E6 TMPRSS2 (120,000 cells per well), Calu-3 (200,000 cells per well), and feline lung cACE2 (250,000 cells per well). Cells were seeded in 12-well plates for 24 h until they reached 80%–90% confluency. Cells were then inoculated with rWA1 and rWA1ΔORF6 (MOI 0.1) and incubated at 4°C for 1 h for virus adsorption. After that, the inoculum was replaced with 1 mL of complete growth media and incubated at 37°C. Cells and supernatants were harvested at 4, 8, 12, 24, 48, and 72 h pi and stored at −80°C. Time point 0 h was an aliquot of virus inoculum stored at −80°C as soon as inoculation was completed. Virus titers were determined in Vero E6 TMPRSS2 cells at each time point using end-point dilutions and the Spearman and Karber’s method and expressed as TCID_50_.mL^–1^.

### Plaque phenotype

The viral plaque phenotype was determined in Vero E6 cells. For this, Vero E6 cells (3 × 10^5^ cells per well) were seeded in 6-well plates for 24 h. Cells were inoculated with rWA1 and rWA1ΔORF6 (10 plaque-forming units [PFU] per well) and incubated at 37°C for 1 h. Following that, the inoculum was removed, and 2 mL of media containing 2× complete growth media and 0.5% SeaKem agarose (final conc. 1× media 0.25% agarose) was added to each well. Once the agarose polymerized, the plate was transferred to the incubator at 37°C for 72 h. The agarose overlay was removed, and the cells were fixed with 3.7% formaldehyde solution for 30 min and stained with 0.5% crystal violet solution for 10 min at room temperature. The plaque size was quantified using a Keyence BZ-X810 Microscope.

### Luciferase reporter assays

The ability of the SARS-CoV-2 ORF6 protein to modulate innate immune pathways was investigated using luciferase reporter assays. Initially, a lentiviral plasmid encoding the SARS-CoV-2 ORF6 protein (pLVX-EF1alpha-SARS-CoV-2-orf6-2xStrep-IRES-Puro, Addgene # #141387) was used in the luciferase reporter assays. For this, HEK293T cells were seeded in 24-well plates at 2 × 10^5^ cells/mL for 24 h and transfected with pIFN-β-Luc, pNF-κB-Luc, pIRF3-Luc, pPRDII-Luc, or pPRDIII-Luc (200 ng/well) and pRN-Luc (50 ng) reporter plasmids in combination with ORF6 protein-expressing plasmid or a pLVX empty vector (250 ng) using Lipofectamine 3000 (Invitrogen, L3000001). At 24 h post-transfection, the cells were stimulated with SeV (Cantell strain, 100 hemagglutination units/well), or TNF-α (25 ng/well), or transfected with 1 µg of poly(I:C) for 12 h. After stimulation, the cells were lysed, and luciferase activity was measured using the dual luciferase assay (Promega, E2940). Luminescence was measured using a luminometer plate reader (BioTek Synergy LX Multimode Reader). The ratio of luminescence, obtained from the target reporters (pIFN-β-Luc, pNF-κB-Luc, pIRF3-luc, pPRDII-luc, or pPRDIII-luc) to luminescence from the control Renilla reporter (pRN-Luc), was calculated to normalize the transfection efficiency. Then, the relative pIFN-β-, pNF-κB-, pIRF3-, pPRDII-, or pPRDIII-driven luciferase activity was calculated as fold changes over the unstimulated cells. Confirmation of the ORF6 expression in HEK293T-transfected cells was performed by western blot using an antibody against Strep-tag II (GenScript).

Furthermore, the luciferase reporter assay was performed using the two recombinant viruses rWA1 and rWA1ΔORF6. For that, HEK293T-hACE2 cells were seeded in 24-well plates (2 × 10^5^ cells/mL). After 24 h, the cells were co-transfected with IFN-β or NF-κB Firefly reporter plasmids (200 ng/well) and the control Renilla reporter (pRN-Luc) plasmid (50 ng/well) for 6 h. Cells were then infected with rWA1 or rWA1ΔORF6 (MOI = 3) for 12 h. After that, the cells were stimulated with SeV (Cantell strain, 100 hemagglutination units/well) or TNF-α (25 ng/well) for 8 h, cell lysates were harvested, and the luciferase activity was measured using a luminometer. Untreated control cells were harvested after 20 h of infection. The ratio of luminescence, obtained from the target reporters (IFN-β-Luc or NF-κB-Luc) to luminescence from the control Renilla reporter pRN-Luc, was calculated to normalize the transfection efficiency. The relative IFN-β- or NF-κB-driven luciferase activity was calculated as fold changes over the unstimulated cells.

To validate the innate immune inhibitory phenotype observed in luciferase reporter assays following ORF6 overexpression, we performed gene expression analysis by quantitative PCR. HEK293T cells (2 × 10⁵ cells per well) were seeded in 12-well plates and cultured for 24 h prior to transfection with plasmids encoding ORF6 or an empty vector control. At 24 h post-transfection, cells were stimulated with Sendai virus (SeV; Cantell strain, 200 hemagglutination units per well), treated with TNF-α (50 ng per well), or transfected with poly(I:C) (2 µg per well) for 4 h. Total RNA was extracted using the RNeasy Mini Kit (Qiagen) with on-column DNase I digestion to remove residual genomic DNA. First-strand cDNA synthesis was performed using the ProtoScript II First Strand cDNA Synthesis Kit (New England Biolabs). Quantitative PCR was carried out using PrimeTime Gene Expression Master Mix (IDT) according to the manufacturer’s instructions. PrimeTime Mini qPCR assays (IDT) targeting human IFNB1 (Hs.PT.58.39481063.g), ISG15 (Hs.PT.58.39185901.g), NFKBIA (Hs.PT.58.15498666.g), and HPRT1 (Hs.PT.58v.45621572) were used for gene expression analysis.

### Animals and housing

A total of 18 domestic cats (*Felis catus*) (nine male and nine female) of 15–18 months of age were obtained from ClinVet (Waverly, NY, USA). The animals were donated to Cornell University to support the reduction of animal use in research. They were housed individually in Horsfall HEPA-filtered cages in the animal biosafety level 3 (ABSL-3) facility at the East Campus Research Facility (ECRF) at Cornell University. Food and water were provided *ad libitum*.

### Experimental design and sample collection

After acclimation, nasal swabs and blood samples were collected from all animals and tested by rRT-PCR for SARS-CoV-2 and virus neutralization using SARS-CoV-2 B.1 and Omicron BA1.1 lineages, which confirmed that the animals were negative for SARS-CoV-2 (27). On day 0, cats were anesthetized and inoculated intranasally with 1 mL virus suspension (0.5 mL per nostril) containing 5 × 10^5^ PFU of SARS-CoV-2 rWA1 (*n* = 6) or rWA1ΔORF6 (*n* = 6). The control animals (*n* = 6) received 1 mL of Vero E6 cell culture supernatant intranasally. Body weight and body temperature were recorded daily until day 5 post-inoculation (pi). Oropharyngeal (OPS) and nasal swabs (NS) were collected under sedation (dexmedetomidine) on days 0, 1, 3, and 5 pi using sterile swabs and placed in 1 mL viral transport medium (VTM Corning, Glendale, AZ, USA) and stored at −80°C until used. Three cats from each group were humanely euthanized on days 3 and 5 pi. Necropsy was performed, and bronchoalveolar lavage fluid, tissues such as nasal turbinate, tonsils, retropharyngeal lymph nodes, trachea, lungs, mediastinal lymph node, heart, liver, spleen, kidney, small intestine, and mesenteric lymph node were collected for rRT-PCR and virus isolation. Besides, tissue sections of approximately 0.5 cm from the nasal turbinate, trachea, and lungs were collected in 10% neutral buffered formalin (≥20 volumes fixative to 1 volume tissue) for histological examination and *in situ* hybridization (ISH). After 72 h of fixing, formalin-fixed tissues were transferred to 80% ethanol and processed for histology.

### Nucleic acid isolation and real-time reverse transcriptase PCR (rRT-PCR)

A 10% (wt/vol) tissue homogenate was prepared using a stomacher (one-speed cycle of 60 s, Stomacher 80 Biomaster). Then, the tissue homogenate supernatant was centrifuged at 2,000 × *g* for 10 min. Viral nucleic acid was extracted from 200 µL of OPS, NS, BALF, and tissue homogenate using the MagMax Core extraction kit (Thermo Fisher, Waltham, MA, USA) and the automated KingFisher Flex nucleic acid extractor (Thermo Fisher, Waltham, MA, USA). The rRT-PCR was performed using the EZ-SARS-CoV-2 Real-Time RT-PCR assay (Tetracore Inc., Rockville, MD, USA) as described earlier (27). An internal inhibition control was included in all reactions. Positive and negative amplification controls were run side-by-side with test samples. A standard curve was performed by using ten-fold serial dilutions from 10^0^ to 10^−8^ of virus suspension containing 10^6^ TCID_50_.mL^−1^ of the SARS-CoV-2 strain. Relative viral genome copy numbers were calculated based on the standard curve and determined using GraphPad Prism 9 (GraphPad, La Jolla, CA, USA). The amount of viral RNA detected in samples was expressed as log (genome copy number) per mL.

### Virus isolation and titrations

The SARS-CoV-2 rRT-PCR-positive samples were selected for virus isolation and endpoint titration under Biosafety level 3 (BSL-3) conditions at the Animal Health Diagnostic Center (ADHC) Research Suite at Cornell University. To this end, Vero E6 TMPRSS2 cells seeded in 96-well plates for 24 h were inoculated with serial 10-fold dilutions of the OPS, NS, BALF, and tissue homogenates. After 48 h, the cells were fixed with 10% neutral buffered formalin, and IFA was performed as described previously (27). The limit of detection (LOD) for infectious virus titration is 10^1.05^ TCID_50_.mL^–1^. Virus titers were determined at each time point using end-point dilutions and the Spearman and Karber’s method and expressed as TCID_50_.mL^–1^.

### *In situ* RNA detection

Formalin-fixed tissues (nasal turbinate, lungs, and trachea) collected from control and SARS-CoV-2-infected cats at days 3 and 5 pi were embedded in paraffin, sectioned at 5 µM, and processed for ISH using the RNAscope ZZ probe technology (Advanced Cell Diagnostics, Newark, CA). The RNAscope 2.5 HD Reagents–RED kit (Advanced Cell Diagnostics) and a probe targeting SARS-CoV-2 RNA S (V-nCoV2019-S probe ref # 848561) were used in the ISH. A probe targeting feline host protein peptidylprolyl isomerase B (PPIB) was used as a positive control (Advanced Cell Diagnostics cat # 455011). A probe targeting the DapB gene from Bacillus subtilis strain SMY was used as a negative control (Advanced Cell Diagnostics cat # 310043). Tissue sections were ISH scored based on labeling extension using a scoring system as follows: score 1 = up to 2% of the tissue section positive; score 2 = from 2% to 5% positive; score 3 = from 5% to 15% positive; score 4 = from 15% to 25%; score 5 = more than 25%; and score 0 = no labeling (27).

### Histology

Formalin-fixed tissues (nasal turbinate, trachea, and lungs) were embedded in paraffin and sectioned at 5 µM diameter following standard procedures. The tissue sections were processed for the routine hematoxylin and eosin (H&E) staining and histological examination. Histological evaluation was conducted by a board-certified veterinary anatomic pathologist (ADM), and tissue alterations were graded for severity using a previously established scoring system (38). In the lungs, lesions in bronchi, bronchioles, alveoli, blood vessels, and pleura were assessed using a combination of severity scores and presence/absence criteria. Lesions were classified as: score 0 (normal = no lesions/physiological levels/absence of inflammatory cells); score 1 (minimal = occasional small foci or incomplete cuffs, affecting ≤5% of the section); score 2 (mild = multiple foci or numerous cuffs, mostly incomplete and loosely arranged, involving 6-25% of the section); score 3 (moderate = widespread lesions across multiple airways, numerous cuffs with about half or more being well-formed, occasionally broad/dense, affecting 26%–50% of the section); and score 4 (severe = extensive lesions across multiple airways, numerous well-formed cuffs with frequent broad dense cuffs, involving >50% of the section). Tracheal and nasal turbinate mucosa, submucosa, and associated vessels were evaluated and scored separately.

### Bulk RNA-seq library prep and sequencing

To understand the effect of ORF6 gene deletion from the wild-type SARS CoV-2 (WA-1) genome on host tissue responses, we performed bulk RNA-seq from nasal turbinate tissue collected at 3 dpi. Total RNA was extracted from the nasal turbinate using an RNA extraction kit (MagMax CORE Extraction), and after on-column DNase treatment, the total RNA integrity score (RIS) was determined with the QIAxcel advanced capillary electrophoresis system (Qiagen), according to the instructions. The NGS cDNA library was prepared by using KAPA RNA HyperPrep Kit with RiboErase Kit (HMR) (Roche, USA) and sequenced with Illumina NovaSeq platform with S1-per lane producing 800 million reads 100bp Single-Reads (Illumina, USA).

### Bulk RNA-seq data prep-processing

The high-quality, clean data from raw reads were obtained by removing reads with adapters and below the Q30 value (FastQC and Trimmomatic). RNA-STAR was used for sequence alignment to the reference cat genome (GCF_000181335.3_Felis_catus_9.0_genom.fasta), and the transcripts were quantified with the annotated cat genome (cat GCF_000181335.3_Felis_catus_9.0_genome.gtf). The obtained reads per gene and features count were used to obtain the gene expression across different groups. DESeq2 was used to compare DEGs in different groups (mock, rWA1∆ORF6, and WA-1). Only the genes with log fold change (logFC) >2, as biologically significant and statistically (*P* < 0.05) significant, were considered DEGs. The heatmap showing the hierarchical clustering of DEGs across different samples and groups was generated to facilitate the comparison of expression profiles between different groups.

### Gene ontology and pathway enrichment analysis

The DEGs in rWA-1, rWA1∆ORF6, and mock groups were used for gene ontology (GO) biological processes (BP), GO molecular functions (MP), and GO cellular responses (CR) using EnrichR bioinformatic resources (https://maayanlab.cloud/Enrichr/). To identify the fold enrichment of specific genes in cell signaling pathways, the statistical overrepresented test (Binomial, *P* < 0.05) was performed by using GCF_000181335.3_Felis_catus_9.0_genom.fasta as reference. The individual bar graphs for pathway enrichment were created using GraphPad Prism software (version 9.0.1).

### Gene expression verification by real-time quantitative PCR (qPCR)

To verify gene expression, total RNA extracted from the nasal turbinate of cats at 3 dpi for RNA-seq study was used in gene verification studies. cDNA was synthesized by ProtoScript II First Strand cDNA Synthesis Kit (NEB). qPCR was performed using PrimeTime Gene Expression Master Mix (IDT) following the manufacturer’s instructions. The following forward and reverse primers and probes were used; IFIH1: forward CACAGGAGTGACTGTCTCATATTC, reverse TTAGACGGCCTCCAAGATTTC, probe TGCCCACACTCAAAGTTCAGGGAT; BST2: forward CCTGCAACAAGACTTTGGTAAC, reverse TGCTTCAACTCCTCAATCTCTC, probe TTCTCCATCTCCAGGGAAGCCAAC; ATF3: forward CTGCAGAAAGAGTCGGAGAAG; reverse CCGATGAAGGTTGAGCATGTA, probe TTCAGTTCGGCATTCACGCTCTCC; TRIM6: forward AAGTGAGTTCTGGACCCTAAAG, reverse GGACATCCGTCAGTTCTCTAAA, probe TGCCCACCAAGCTGAAGAGTATGT; IDO1: forward TGACCTCAAAGACCACAAGTC, reverse TGGCAAGACCTCACAAACA, probe TATGTGTGGAACCAAGGCGGTGAA; SAMHD1: forward TGTGGAATGGACGCATGAA, reverse CTTCAGGGATGAGACCGTAATG, probe AGGGCTCAGTGAAGATGTTTGAGCA; S100A9: forward GCATTGAGACCATCATCAACATC, reverse CACCAGCTGTTTCAGTTCTTTC, probe TGGAGCACCCGGACAAACTCAA; IFIT2: forward CTATGGCAACTTCCAGCTCTAC, reverse GTCTCCTTTGAGTCCTGCTTTAT, probe CGAGGACAGAGCCATCCACCATTT; RSAD2: forward TGGTGAGGTTCTGCAAAGAG, reverse AGATGGCAAGGATGTCCAAATA, probe CGCTGGTTCAGGACTTACGGTGAA; and GAPDH: forward AAGGCTGAGAACGGGAAAC, reverse ATACTCAGCACCAGCATCAC, probe TGGAAAGCCCATCACCATCTTCCA. All primers were first tested for their specificity and efficiency using serial 5-fold dilutions of cDNA prepared from pooled nasal turbinate RNA of SARS-CoV-2-inoculated cats.

### Statistical analysis and data plotting

Statistical analysis was performed using two-way analysis of variance (ANOVA), followed by multiple comparisons. Statistical analysis and data plotting were performed using GraphPad Prism software (version 9.0.1). Figures 1A and 4A were created using BioRender.com.

## Data Availability

All data pertaining to this study are presented in the article.
